# ^68^Ga-DOTATATE PET/CT Compared with ^131^I-MIBG SPECT/CT in the Evaluation of Neural Crest Tumors

**DOI:** 10.22038/aojnmb.2019.41343.1280

**Published:** 2020

**Authors:** Pezhman Shahrokhi, Alireza Emami-Ardekani, Sara Harsini, Mohammad Eftekhari, Armaghan Fard-Esfahani, Babak Fallahi, Najme Karamzade Ziarati, Mehdi Akhlaghi, Saeed Farzanefar, Amir Pejman Hashemi Taheri, Davood Beiki

**Affiliations:** 1 Research Center for Nuclear Medicine, Shariati Hospital, Tehran University of Medical Sciences, Tehran, Iran; 2 Department of Nuclear Medicine, Vali-Asr Hospital, Tehran University of Medical Sciences, Tehran, Iran; 3 Department of Radiology, Shariati Hospital, Tehran University of Medical Sciences, Tehran, Iran; † These authors shared first authorship

**Keywords:** ^68^Ga-DOTATATE, ^ 131^I-MIBG, PET/CT, Neural crest tumors

## Abstract

**Objective(s):**

^68^Ga-DOTATATE positron emission tomography (PET)/computed tomography (CT) has shown promising results in imaging of neural crest tumors (NCT). Herein, we compared the performance of ^68^Ga-DOTATATE PET/CT and ^131^I-MIBG single photon emission computed tomography (SPECT)/CT in the initial diagnosis, staging and follow-up of patients with NCTs.

**Methods:**

Twenty-five patients (males:females=8:17; age range=2–71 years) with clinically proven or suspicious neuroblastoma, pheochromocytoma (PCC) or paraganglioma (PGL) were enrolled in this prospective study and underwent both ^68^Ga-DOTATATE PET/CT and ^131^I-MIBG SPECT/CT. A composite reference standard derived from histopathological information, together with anatomical and functional imaging findings, was used to validate the results. Imaging findings were assessed on a per-patient and on a per-lesion basis. Sensitivity and accuracy were assessed using McNemar’s test.

**Results:**

Referring to radiological imaging and histopathological findings as reference standard, ^68^Ga-DOTATATE and ^131^I-MIBG scans showed a sensitivity and accuracy of (100%, 96%) and (86.7%, 88%), respectively, on a per-patient basis. In PCC/PGL patients, on a per-patient basis, the sensitivity of ^68^Ga-DOTATATE was 100% and that of ^131^I-MIBG was 77.8%. In neuroblastoma patients, on a per-patient basis, the sensitivities of both ^68^Ga-DOTATATE and ^131^I-MIBG were 100%. Overall, in this patient cohort, ^68^Ga-DOTATATE PET/CT identified 52 lesions and ^131^I-MIBG SPECT/CT identified only 30 lesions. On a per-lesion analysis, ^68^Ga-DOTATATE was found to be superior to ^131^I-MIBG in detecting lesions in all anatomical locations, particularly osseous lesions. According to the McNemar test results, differences were not statistically significant.

**Conclusion:**

This relatively small patient cohort suggests ^68^Ga-DOTATATE PET/CT be superior to ^131^I-MIBG SPECT/CT in providing particularly valuable information for both primary staging and follow-up in patients with NCT.

## Introduction

 Neuroblastomas (NBs), ganglioneuroblastomas, pheochromocytomas (PCCs) and paragangliomas (PGLs) are acknowledged as neural crest tumors (NCTs), deriving from the sympathetic nervous system, which itself comprises collections of neuroepithelial cells scattered throughout the body, anywhere from the neck to the pelvis, containing numerous neurosecretory granules of catecholamines as their definitive common feature. The better differentiated tumors arising from this system are known as paragangliomas, containing numerous catecholamine granules. Paragangliomas of the adrenal medulla, believed to arise from chromaffin-producing cells, are regarded as pheochromocytomas. On the other hand, poorly differentiated tumors arising from precursors of this system are known as neuroblastomas (1). These tumors of neuroendocrine origin mainly produce catecholamines and related substances, resulting in their increased plasma and urinary levels. Metastases originating from the malignant forms of this group of tumors could be identified in soft tissue, bone, bone marrow, lymph nodes and in other organs, namely, liver and lung. 

 The diagnosis and follow-up of NCTs is accomplished by measuring the level of serum/urine tumor markers (catecholamines, metanephrines) together with cross-sectional and functional imaging methods, providing anatomic information and predicting tumor localization and extent (2). Computed tomography (CT) and magnetic resonance imaging (MRI) are the morphologic imaging modalities of choice in localizing these tumors with high sensitivity in lesion detection, but lack of appropriate specificity due to the difficulties in distinguishing between tumors deriving from the sympathetic nervous system or other tumor entities (2, 3). Although these imaging methods are often regarded as first-line imaging modalities, functional imaging exerts advantages of high sensitivity, very high specificity, and performance of whole-body scanning, and are thus commonly suggested to clarify equivocal lesions detected by anatomical imaging. 

^123/131^Iodine-metaiodobenzylguanidine (^123/131^I-MIBG) is currently contemplated as the reference tracer for single photon emission computed tomography (SPECT). In addition, SPECT imaging for somatostatin receptor (SSTR) has been widely carried out in neuroendocrine tumors (NETs) and also in NB, using somatostatin analogue ^111^In-pentetreotide (4). Considering the shortcomings of SPECT imaging, including its limited sensitivity and spatial resolution, and the long duration of image acquisition, positron emission tomography (PET) is becoming the leading functional modality in cancer imaging, on the basis of its better image resolution, shorter duration of the procedure, and more accurate quantification. A few clinical studies have described PET imaging in human NCTs, using metabolic tracers such as the glucose analogue ^18^F-fluorodeoxyglucose (FDG) and the amino acid analogue ^18^F-fluorodopa (5, 6). PET imaging of SSTR could be performed using somatostatin analogues such as ^68^Ga-[tetraxetan-D-Phe1, Tyr3]-octreotate (^68^Ga-DOTATATE), a somatostatin analogue suited for PET imaging displaying very high SSTR2 receptor binding. 

 Herein we report a cohort of patients with NCTs and contemporaneous ^131^I-MIBG SPECT/CT and ^68^Ga-DOTATATE PET/CT imaging. The aim was to compare the performance of these functional imaging modalities in detecting malignant PCC, PGL and NB in the initial diagnosis and follow-up of these patients.

## Methods

***Study population***

 Twenty-five consecutive patients (males: females=8:17; age range 2–71 years; median age of 8 years) who underwent both ^68^Ga-DOTATATE PET/CT and ^131^I-MIBG SPECT/CT examinations either for primary diagnosis, initial staging, restaging, evaluation of metastases, evaluation of response to treatment, and surveillance of NCTs were recruited in this prospective study (Table 1). Among them, twenty-four patients had biochemically and histologically proven NCTs (fifteen neuroblastoma, eight PCCs, and one PGL) and one patient was diagnosed with von Hippel–Lindau disease (VHL) and was suspected to have PCC. Patients who met the following criteria were included in the investigation: (a) raised 24-h urine catecholamine, (b) persistent uncontrolled blood pressure while on more than two anti-hypertensive medications, (c) histologically proven metastatic disease for further restaging, (d) suspicion of metastasis according to the presence of suspicious metastatic lesion(s) on contrast-enhanced CT or MRI, and (e) primary adrenal mass of greater than 5 cm. All examinations took place within 30 days from each other with no therapeutic interventions between the two examinations. 

**Table 1:** Characteristics of patients with neural crest tumors and details of tracer uptake in ^68^Ga-DOTATATE PET/CT and ^131^I-MIBG SPECT/CT

**Patient no.**	**Gender**	**Age years**	**History**	**Indication**	**Lesion(s) detected** **with ** ^131^ **I-MIBG**	**Lesion(s) detected with ** ^68^ **Ga-DOTATATE**	**Size of lesion(s) (mm)**
1	F	16	Neuroblastoma	Surveillance	0	0	-
2	F	55	Pheochromocytoma	Staging	Bone,1; mediastinum/lung,3; abdominopelvic, 2	Mediastinum/lung,3; abdominopelvic, 2	-
3	F	13	Paraganglioma	Staging	Mediastinum/lung,1; abdominopelvic, 1	Cervicothoracic,1; abdominopelvic, 1	53, 37
4	F	52	Neuroblastoma	Staging	Abdominopelvic, 2	Abdominopelvic, 1	107, 24
5	F	5	Neuroblastoma	Restaging	Bone, 7; abdominopelvic, 5	Bone,3;neck,1; abdominopelvic, 1	Range from 7.3 to 17.5
6	M	45	Pheochromocytoma	Restaging	Bone, 5; abdominopelvic, 1	Bone,2; abdominopelvic, 2	Range from 8 to 15.2
7	F	4	Neuroblastoma	Restaging	Bone,1;neck,1, mediastinum/lung, 1	Cervicothoracic, 1	Range from 9 to 42.6
8	F	3	Neuroblastoma	Restaging	0	0	-
9	F	6	Neuroblastoma	Restaging	0	0	-
10	M	2	Neuroblastoma	Restaging	0	0	-
11	F	16	Pheochromocytoma	Evaluation of metastasis	Neck, 1; abdominopelvic, 2	Neck1; abdominopelvic, 1	32.5, 14.4, 49
12	M	4	Neuroblastoma	Evaluation of metastasis	Mediastinum/lung, 1	Cervicothoracic, 1	14.2
13	F	3	Neuroblastoma	Evaluation of metastasis	Bone,1; abdominopelvic, 2	Abdominopelvic, 2	14.3, 37.5, 28
14	F	41	Pheochromocytoma	Evaluation of metastasis	Abdominopelvic, 2	Abdominopelvic, 2	18.3, 60.2
15	M	14	Pheochromocytoma	Evaluation of metastasis	Abdominopelvic, 3	Abdominopelvic, 1	10, 10, 40
16	M	44	Pheochromocytoma	Evaluation of metastasis	Abdominopelvic, 1	Abdominopelvic, 1	51.7
17	F	42	Pheochromocytoma	Evaluation of metastasis	Mediastinum/lung, 2	0	10, 14
18	M	6	Neuroblastoma	Evaluation of metastasis	0	0	-
19	M	7	Neuroblastoma	Evaluation of metastasis	0	0	-
20	F	6	Neuroblastoma	Evaluation of metastasis	0	0	-
21	F	5	Neuroblastoma	Evaluation of response to treatment	Abdominopelvic, 1	Neck,1; abdominopelvic, 1	44
22	F	8	Neuroblastoma	Evaluation of response to treatment	0	0	-
23	F	2	Neuroblastoma	Evaluation of response to treatment	0	0	-
24	F	22	Von Hippel-Lindau suspected pheochromocytoma	Diagnosis	Abdominopelvic, 3	Abdominopelvic, 1	12, 20, 55
25	F	71	Pheochromocytoma	Diagnosis	Abdominopelvic, 2	0	14, 16

 If necessary, young children were sedated to allow imaging. None of the patients was on medication known to interfere with the cellular uptake of catecholamines, comprising sympathomimetic amines, tricyclic antidepressants, or reserpine (7). It has been an interval of more than 4 weeks between the last injection of long-acting SST analogues and performance of ^68^Ga-DOTATATE PET/CT scans. The study protocol was approved by the Ethics Committee of Tehran University of Medical Sciences and written informed consent was obtained from all patients or their guardians.

***Radiopharmaceutical preparation***

 Ga-68 was obtained from a commercially ^68^Ge/^68^Ga (PARS-GalluGEN) generators, which is locally available in the country form Pars Isotope Company, Tehran, Iran. ^131^I-MIBG and DOTA-Tyr^3^-Octreotate (DOTATATE) kit were also provided from this company. The radiopharmaceutical preparation (^68^Ga-DOTATATE) and all quality controls were carried out according to the standard protocols provided by the kit manufacturer.

^68^ ***Ga-DOTATATE PET/CT imaging protocol***

 PET imaging was performed using Biograph 6 PET/CT scanner (Siemens Medical Solutions, Erlangen, Germany). Depending on the weight of the patients, 4-5 mCi of ^68^Ga-DOTATATE was administered intravenously. Image acquisition was performed at 60-min post-injection with a whole-body field of view (vertex to mid-thigh), and patients were asked to empty their bladder. The CT exposure factors for all examinations were 80 mAs, 80-130 keV, pitch of 0.8 and slice thickness of 5 mm. maintaining patient position, a whole-body PET scan was performed immediately after CT acquisition and covered an area identical to that covered by CT. PET acquisition was carried out in 3D with 4 min per bed position. PET images were reconstructed using CT for attenuation correction. Using ordered subsets expectation maximization algorithm (four iterations and eight subsets), transaxial PET data were reconstructed.

^131^ ***I-MIBG SPECT/CT imaging protocol***

 To minimize ^131^I uptake in the thyroid gland, 20 drops of Lugol’s solution twice a day were administered orally 3 days prior to injection of ^131^I-MIBG and continued 7 more days after the procedure. In young children, the administration of Lugol’s solution was adapted to body weight. A dose of 37-45 MBq (1.0–1.2 mCi) of ^131^IMIBG was injected intravenously. Standard whole-body planar images (simultaneous anterior and posterior views, 256×256 matrix, 5 cm/min) was carried out at 24 h and 72 h using a Siemens Symbia T1 gamma camera with a high-energy parallel-hole collimator with the patient supine. Using 120 projections (60 views x dual heads) at 25-35 s per view, SPECT of the cervicothoracic and abdominopelvic regions were performed over a 180° arc (clockwise rotation) and images were obtained as 128×128 matrix at a zoom factor of 1, using a step-and-shoot rotation with an auto-continuing non-circular orbit. Low dose CT was performed with the 72-h post injection acquisition for attenuation correction and anatomic orientation. SPECT images without (24 h after injection) and with attenuation correction (72 h after injection) were reconstructed iteratively.

***Image interpretation***

 Two experienced nuclear medicine physicians who were aware of the patient’s clinical history but blinded to any results of the anatomical imaging modalities, reviewed all ^68^Ga-DOTATATE PET/CT and ^131^I-MIBG SPECT/CT images on a Mac-based OsiriX workstation and an ESOFT Syngo workstation (Siemens Healthcare), respectively. The site and number of lesions were examined. A per-lesion and per-patient analysis were executed for both imaging modalities. If the results acquired by the two viewers were discordant, a third reader was consulted as a referee. Positive uptakes on PET findings were based on visual assessment and maximum standardized uptake values (SUV_max_) with correction for body weight. Clear demarcation of the lesion with tracer accumulation higher than that of the liver and tracer uptake greater than physiological activity was implemented as the criteria for interpreting a scintigraphic lesion as malignant. ^131^I-MIBG uptake in the adrenals was considered to be normal if it was mild, symmetrical and not enlarged. Both higher and distinct focus of increased tracer uptake from the background and from MIBG normal distribution or side asymmetry of the adrenal glands were considered pathological on MIBG images (8). All patients underwent further morphologic imaging using contrast-enhanced CT, MRI, or ultrasound during their clinical work-up.

 On a per-patient basis, scans were assumed to be positive provided that at least one malignant lesion (primary tumor and/or metastases) was seen, regardless of the number of foci, while, scans with no, mild or symmetrical uptake were considered to be negative. A composite reference standard was adopted to validate the findings by incorporating both histological proof of metastatic lesions together with anatomical and functional imaging information. At least one tumor site was confirmed histologically in all patients investigated. In patients with multiple tumor manifestation, not all sites were verified histologically because of the advanced stage of the disease and systemic therapy as the first therapeutic choice. Morphologic imaging methods and the clinical and imaging follow-up were used so as to clarify discrepant results between ^68^Ga-DOTATATE PET/CT and ^131^I-MIBG SPECT/CT. Clinical and imaging follow-up was considered in case of clearly positive PET/CT or SPECT/CT but negative morphologic imaging. On the other hand, if either PET/CT or SPECT/CT was negative in a region with a clearly visible lesion in morphologic imaging, the negative finding was assumed to be false-negative.

 Analysis on a per-lesion basis was performed on the following areas: cervicothoracic soft tissue, abdominopelvic soft tissue, and bone. For sensitivity calculations, studies were considered either positive or negative. 

***Statistical analysis***

 Normal distribution was assessed by the Kolmogorov–Smirnov test. In the per-lesion analysis, histopathologically positive results or unequivocal focus/foci of abnormally increased tracer uptake detected by either ^68^Ga-DOTATATE PET/CT or ^131^I-MIBG SPECT/CT imaging in a nonphysiological site, confidently judged as disease by the reporting physicians were considered to be true positive. In cases in which the lesion detected by contrasted/non-contrasted CT or MRI showed no uptake on either ^68^Ga-DOTATATE PET/CT or ^131^I-MIBG SPECT/CT imaging was considered true-negative. The presence of at least one positive lesion per patient was assumed to be a positive finding for metastatic disease in the per-patient analysis.

 SPSS Software (version 24.0) was used to perform statistical analysis. Nonparametric methods were used. Sensitivity, specificity, positive predictive value (PPV), negative predictive value (NPV), and accuracy were calculated. The McNemar test was used to compare sensitivities, specificities, PPVs, NPVs and accuracies between the different diagnostic modalities. The number of lesions per patient was compared using the Wilcoxon signed-rank test. One-way analysis of variance (ANOVA) was also carried out. A p value of less than 0.05 was considered statistically significant.

## Results

***Per-patient analysis***

 We compared the two modalities of functional imaging on a per-patient basis. Of the 25 cases in the series, 14 were concordant with both modalities picking up clinically significant lesions, although, one out of these 14 patients (patient number 24) who was initially suspected to have hepatic metastasis was subsequently found to have hepatic hydatid cyst on both US and MRI, causing false positive interpretation of both ^68^Ga-DOTATATE PET/CT and ^131^I-MIBG SPECT/CT images. In addition, nine cases were concordant with both modalities showing no clinically significant lesion, determining an appropriate response to treatment. There were no patients in whom both modalities failed to pick up clinically significant lesions. There was discordance in two patients having positive ^68^Ga-DOTATATE and negative ^131^I-MIBG (Table 2). Utilizing further anatomical imaging modalities and histopathological studies as the gold standard, the sensitivity, specificity, accuracy, positive predictive value, and negative predictive value of ^68^Ga-DOTATATE PET/CT and ^131^I-MIBG SPECT/CT were (100%, 90%, 96%, 93.7%, 100%) and (86.7%, 90%, 88%, 93.7%, 81.8%), respectively. No statistically significant differences were demonstrated by the McNemar test.

 A per-patient analysis was performed to compare the diagnostic performance of ^68^Ga-DOTATATE PET/CT and ^131^I-MIBG SPECT/CT for anatomical sites of the lesions detected, as well as groups of patients with neuroblastoma and PCC/PGL, separately (Table 3). McNemar test revealed no statistically significant differences. The greatest discordance was in the detection of osseous lesions, where 5 patients were picked up with ^68^Ga-DOTATATE but ^131^I-MIBG picked up 2.

**Table 2:** Per-patient and per-lesion comparison of scans

		**MIBG-positive**	**MIBG-negative**	**Total**
	**DOTATATE-positive**	14	2	16
**Per-patient analysis**	**DOTATATE-negative**	0	9	9
	**Total**	14	11	25
	**Cervicothoracic**	10		
**Per-lesion analysis (DOTATATE-positive)**	**Abdominopelvic**	27		
	**Bone**	15		
	**Cervicothoracic**	9		
**Per-lesion analysis (MIBG-positive)**	**Abdominopelvic**	16		
	**Bone**	5		

Per-lesion analysis has been carried out according to anatomic distribution of the lesions.

**Table 3:** Per-patient analysis of scans based on tumor histology and the anatomical regions

**Imaging Modality**	**Tumor Histology/Localization**	**TP**	**FP**	**TN**	**FN**	**Sensitivity**	**Specificity**	**PPV**	**NPV**	**Accuracy**
^68^ **Ga-DOTATATE PET/CT**	Neuroblastoma	6	0	9	0	100%	100%	100%	100%	100%
	PCC/PGL	9	1	0	0	100%	N/A	90%	N/A	90%
	Cervicothoracic region	6	0	18	1	85.7%	100%	100%	94.7%	96%
	Abdominopelvic region	12	1	11	1	92.3%	91.7%	92.3%	91.7%	92%
	Bone	5	0	20	0	100%	100%	100%	100%	100%
^131^ **I-MIBG SPECT/CT**	Neuroblastoma	6	0	9	0	100%	100%	100%	100%	100%
	PCC/PGL	7	1	0	2	77.8%	N/A	90%	N/A	70%
	Cervicothoracic region	6	0	18	1	85.7%	100%	100%	94.7%	96%
	Abdominopelvic region	13	1	11	0	100%	91.7%	92.9%	100%	96%
	Bone	2	0	20	3	40%	100%	100%	86.9%	88%

PCC/PGL, pheochromocytoma/paraganglioma; TP, true positive; FP, false positive; TN, true negative; FN, false negative; PPV, positive predictive value; NPV, negative predictive value

***Per-lesion analysis***

**Figure 1:**
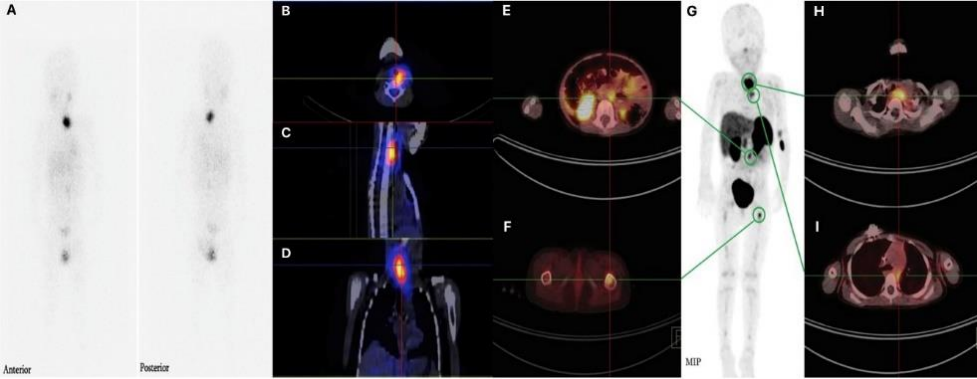
^131^I-MIBG and ^68^Ga-DOTATATE images of a 3-year-old boy with neuroblastoma, taken less than 1 month apart. (A) ^131^I-MIBG whole-body scintigraphic images, together with (B) transverse, (C) sagittal, and (D) coronal SPECT/CT images show a metastatic lesion in the cervical paravertebral region. (G) Maximal intensity projection ^68^Ga-DOTATATE PET image, along with ^68^Ga-DOTATATE PET/CT fusion images (E, F, H, I) depict metastatic lesions in the cervical and mediastinal paravertebral regions, retroperitoneum, and proximal part of the left femur. The lesions demonstrated on ^68^Ga-DOTATATE images clearly outnumber ^131^I-MIBG images

 A total of 54 lesions of variable sizes ranging from 7.3 to 60.2 mm were detected when considering both ^131^I-MIBG and ^68^Ga-DOTATATE findings. ^68^Ga-DOTATATE located 52 lesions, whereas ^131^I-MIBG detected fewer lesions at 30 (Tables 1 and 2). Three of the lesions detected on ^68^Ga-DOTATATE and one lesion observed on ^131^I-MIBG were attributed to hepatic hydatid cyst and thus interpreted as false positive. The range of measured SUV_max_ over ^68^Ga-DOTATATE uptake lesions was from 1.99 to 27.70 with a mean of 8.74±6.42. As we had no reference to check every single lesion as either true or false positive or negative, sensitivity and accuracy of these two modalities could not be assessed. As it is evident by Table 1 and 2, ^68^Ga-DOTATATE detected more lesions than ^131^I-MIBG across all anatomical locations (Figure 1). This was particularly so with bony lesions where a total of 15 lesions were detected by ^68^Ga-DOTATATE compared to just 5 by ^131^I-MIBG (Table 2). The number of lesions per patient in ^68^Ga-DOTA-TATE PET/CT and ^131^I-MIBG SPECT/CT suggested the superiority of the former modality in detecting metastatic lesions using the Wilcoxon signed-rank test (P value=0.004).

A correlation between lesions’ size and their detectability on ^131^I-MIBG scan was evident by one-way ANOVA test (P value=0.004). It has been shown that the mean size of lesions detected merely on PET/CT but not on the MIBG scan was 14.4±8.8 mm. However, no clear correlation between SUV_max_ of the lesions observed on ^68^Ga-DOTATATE PET/CT and their detectability on ^131^I-MIBG scan was evident.

## Discussion

 In spite of the well-known limitations of MIBG scintigraphy, including the complicated patient preparation (including thyroid blockade and discontinuation of certain drugs), low spatial resolution, prolonged imaging protocol, physiological uptake restricting in detection of small lesions, and relatively high radiation dose to the patients, this imaging method has been regarded as a standard functional imaging for initial evaluation and management of a subgroup of NCTs, namely, PCC, PGL, and neuroblastoma (9). Notwithstanding the fact that ^123^I-MIBG is considered as the standard in clinical practice due to the favorable characteristics of the labelling radioisotope ^123^I, with its shorter half-life, lack of beta particulate emission, and higher emitted photon flux (10) as compared to ^131^I, ^123^I might not be available in every facility, and this results in the administration of ^131^I, which suffers from low image quality and unfavorable dosimetry, instead. With the advent of the PET imaging technique and radiolabeling of somatostatin analogues with positron emitting isotopes, including ^68^Ga, for imaging somatostatin receptor expressing tumors, ^68^Ga-DOTATATE PET scan has demonstrated dramatic improvement of the spatial resolution and lesion detectability compared to MIBG scintigraphy (11). Additionally, ^68^Ga-DOTATATE PET imaging exhibits both practical advantages (no patient preparation, easy synthesis, short and convenient (less than 2 hours) imaging procedure, lower radiation exposure (12) and wide availability due to ^68^Ge/^68^Ga generator), and added diagnostic information and quantification capability. This imaging modality has also been proven to have high impact on management of patients with neuroendocrine cancers, signifying its role in clinical practice of somatostatin-avid malignancies (13). In spite of the speculated superior diagnostic performance of PET with various radiopharmaceuticals in comparison with MIBG scintigraphy, based on cumulative official and clinical experiences, MIBG is still considered as the workhorse in evaluation of NCT. A potential drawback of ^68^Ga-DOTATATE PET imaging could be the high cost of ^68^Ge/^68^Ga generators. However, recent approval of the SST analogue kit by the United States Food and Drug Administration along with the increasing demand for ^68^Ga-labelled radiotracers will make ^68^Ge/68Ga generators more readily available. In addition, imaging centralization and a referral system, as examples of an effective systematic planning, would help reduce the cost of ^68^Ga imaging.

 Herein, we described 25 patients with neuroblastoma, PCC, and PGL with comparative ^131^I-MIBG SPECT/CT and ^68^Ga-DOTATATE PET/CT evaluations. This investigation aims to add to the recent small case series comparing ^131^I-MIBG and ^68^Ga-DOTATATE in the diagnosis and follow-up of NCTs. In line with previous studies (14, 15), the observed diagnostic performance of ^131^I-MIBG scintigraphy in the detection of metastatic PCC/PGL on per-patient and per-lesion basis was rather disappointing, although this was not the case with neuroblastoma. As ^131^I-MIBG scintigraphy is found to remarkably underestimate the disease extent, its use as a diagnostic agent should be confined to evaluation of patient’s eligibility for ^131^I-MIBG therapy (16). Utility of ^131^I-MIBG SPECT/CT has been advocated to increase the diagnostic certainty in indeterminate lesions, and better fair situation for comparison with ^68^Ga-DOTATATE images, improving diagnostic performance compared to planar imaging (17). Findings of the current study demonstrated marginally better sensitivity and accuracy of ^68^Ga-DOTATATE PET/CT compared to than ^131^I-MIBG SPECT/CT on per-patient and per-lesion bases, however, we could not detect any statistically significant superiority of ^68^Ga-DOTATATE PET/CT over ^131^I-MIBG SPECT/CT. Anatomical areas have been assumed to affect the detection rate in functional imaging studies (18). In our study, ^68^Ga-DOTATATE identified an equal or greater number of patients with disease than ^131^I-MIBG in all anatomical areas (Table 2). The per-lesion comparison further revealed the impact of the anatomical distribution of disease on pick-up rate. ^68^Ga-DOTATATE showed better sensitivity in all anatomical positions than ^131^I-MIBG, except for the lesions in the abdominopelvic region. The superior diagnostic performance of ^68^Ga-DOTATATE imaging was particularly noticeable for osseous lesions, in which ^68^Ga-DOTATATE detected nearly threefold more lesions than ^131^I-MIBG (15 vs. 5). This could be partly attributed to the smaller size of bony lesions, which are better distinguished by PET/CT due to its higher spatial resolution than SPECT/CT.

 Win et al. were the first to analyze and compare ^68^Ga-DOTATATE and MIBG imaging in small group of patients with neuroectodermal tumors (19). They found ^68^Ga-DOTATATE capable of identifying more lesions with higher uptake and better resolution compared to ^123^I-MIBG. Out of 5 patients, two had negative ^123^I-MIBG and positive ^68^Ga-DOTATATE scans, one had a weakly positive ^123^I-MIBG and a strongly positive ^68^Ga-DOTATATE scan, and one had a positive ^123^I-MIBG and positive ^68^Ga-DOTATATE scans. Similar finding was noted in the study by Naji et al, suggesting ^68^Ga-DOTATATE PET to be superior to ^123^I-MIBG SPECT, as ^68^Ga-DOTATATE PET could detect tumor lesions in ten out of 12 patients with confirmed disease, while ^123^I-MIBG depicted lesions in five out of 12 patients (20). These two imaging methods detected a total of 30 lesions, of which 29/30 was positive with ^68^Ga-DOTATATE and 7/30 with ^123^I-MIBG. Maurice et al. evaluated the performance of ^68^Ga-DOTATATE PET/CT and ^123^I-MIBG SPECT, and observed that in head and neck tumors in 4 patients, lesions were picked up by ^68^Ga-DOTATATE, but missed by ^123^I-MIBG (21). ^68^Ga-DOTATATE was able to detect 167 lesions versus 111 lesions detected by MIBG imaging and 128 detected by CT/MRI, on a per-lesion analysis. Their results divulged the superiority of ^68^Ga-DOTATATE in detecting lesions in all anatomical locations, particularly osseous metastases, and suggested ^68^Ga-DOTATATE as a first-line investigation in patients at high risk of PGL and metastatic disease, and as the preferred imaging modality in patients with metastatic spread, particularly if the osseous involvement is suspected. In another study performed by Tan et al, which eventually found ^68^Ga-DOTATATE PET/CT, having higher diagnostic accuracy than ^131^I-MIBG scintigraphy and ^18^F-FDG PET/ CT in mapping metastatic PCC/PGL, 17 patients with clinically proven or suspicious metastatic PCC/PGL were evaluated; 12 patients by ^68^Ga-DOTATATE PET/CT, ^18^F-FDG PET/CT and ^131^I-MIBG scintigraphy and five by merely ^68^Ga-DOTATATE and ^131^I-MIBG without ^18^F-FDG (15). A composite reference standard derived from anatomical and functional imaging findings, together with histopathological information, was utilized to validate the findings. 14/17 patients were detected in ^68^Ga-DOTATATE and 7/17 patients in ^131^I-MIBG, on a per-patient basis. The sensitivity and accuracy of ^68^Ga-DOTATATE and ^131^I-MIBG were (93.3 %, 94.1 %) and (46.7 %, 52.9 %), respectively. On a per-lesion basis, an overall of 472 positive lesions were seen; of which 432/472 were recognized by ^68^Ga-DOTATATE and 74/472 by ^131^I-MIBG. The sensitivity and accuracy of ^68^Ga-DOTATATE and ^131^I-MIBG were (91.5 %, 92.6 %, p<0.0001) and (15.7 %, 26.0 %, p<0.0001), respectively. In another investigation, Jing et al. analyzed 8 patients suspected of having primary PCC/PGL and 1 patient with known PCC by ^68^Ga-DOTATATE PET/CT, FDG PET/CT, and MIBG SPECT/CT and compared the imaging findings with postsurgical pathology and follow-up (22). Both Ga-DOTATATATE PET/CT and MIBG SPECT/CT correctly identified 9 primary tumors. Ga-DOTATATATE PET/CT was found to be able to detect associated extra-adrenal lesions not revealed by MIBG study in patients with multiple endocrine neoplasia syndrome. They suggested Ga-DOTATATA PET/CT as the nuclear medicine imaging of choice to evaluate suspected primary PCC/PGL, specifically in the situation of multiple endocrine neoplasia syndrome. Results of these previously performed investigations seem to suggest the diagnostic superiority of ^68^Ga-DOTATATATE PET/CT over ^123/131^I-MIBG scintigraphy in patients with NCTs; nevertheless, discordant findings using these radiopharmaceuticals are also reported.

 It is evident that accurate mapping of all metastatic lesions could have potential implication in assignment of cancer staging, subsequent guidance in therapeutic approach, and finally as an important indicator in prognostication. Underestimation of tumor extent could potentially culminate in inappropriate management, particularly when choosing the appropriate mode of therapy between high risk metastasectomy, palliative chemotherapy or targeted radiotherapy. Moreover, comprehensive evaluation of all target lesions is required to assess therapeutic response, properly (23). The higher diagnostic performance of ^68^Ga-DOTATATE PET/CT, on a per-lesion basis, in comparison with ^131^I-MIBG scintigraphy may somehow have a potential impact on therapeutic decision making. Surprisingly, despite the poorer inherent characteristics of ^131^I than ^123^I for imaging with current gamma cameras, which contribute to poorer target-to-background ratio, our observed sensitivity of ^131^I-MIBG scintigraphy in detecting metastatic NCTs is substantially higher than what was reported in other studies which used ^123^I-MIBG scintigraphy (14, 24-26). Discrepancies observed in our results from those previously reported in other studies may be either related to the utilization of SPECT/CT for better characterization of the lesions, or the use of different reference standards in verifying the disease existence. In the current investigation, a composite reference standard derived from anatomical and functional imaging findings, together with histopathological information, has been adopted to overcome the imperfect standards of some of the previous studies, in which, CT or MRI, generally having low specificity and greater number of inconclusive results, particularly for small lesions, were served as the reference standard. 

 This study has certain limitations that are required to be acknowledged. Firstly, our relatively small sample size, although justified by the rarity of the disease, hinders further analysis. Another limitation was our inability to perform genetic analysis so as to assess potential relationship between ^68^Ga-DOTATATE PET/CT and ^131^I-MIBG SPECT/CT results and genetic abnormalities, leading to a more comprehensive understanding of the correlation between germline mutation and tumor characteristics. As the justification of the diagnostic performance of an index test could be a strenuous task when the gold standard is imperfect, a composite reference standard has been adopted to produce more meaningful results in clinical practice; however, the possibility of reference-standard-related bias, comprising error and variation in image interpretation and misclassification of lesions, cannot be totally excluded.

 Notwithstanding the fact that disease extension in NCTs could be accurately examined by a multimodality approach using different radiotracers, this approach is time-consuming, impractical and not cost-effective. The marginally higher sensitivity and accuracy of ^68^Ga-DOTATATE PET/CT compared to^131^I-MIBG, implies that somatostatin receptor PET/CT imaging may offer a better overview on tumor spread and extension and recommends ^68^Ga-DOTATATE PET/CT into the standard algorithm of evaluation of such tumors. In cases with a high pretest probability of metastatic NCTs, ^68^Ga-DOTATATE PET/CT should be considered as the preferred investigation to exclude active disease, when ^131^I-MIBG scintigraphy does not detect lesions. In addition, ^68^Ga-DOTATATE PET/CT should be considered in preference to ^131^I-MIBG scan in NCTs when metastatic spread, particularly to the bone, is questioned. Moreover, as the high detectability of somatostatin receptor expression in metastatic NCTs provides the basis for consideration of peptide receptor radionuclide therapy (PRRT) as part of the multimodality management of this group of malignancies, ^68^Ga-DOTATATE PET/CT, providing valuable information on somatostatin receptor status in this group of patients. Bearing all the above-mentioned advantages of ^68^Ga-DOTATATE PET/CT in mind, further large prospective and multicenter studies comparing this modality with MIBG SPECT/CT in this setting are required.

## Conclusion

^68^Ga-DOTATATE PET/CT could be superior to ^131^I-MIBG SPECT/CT in providing particularly valuable information for both primary staging and follow-up in patients with neural crest tumors.
